# Modulation of myeloid-derived suppressor cell functions by oral inflammatory diseases and important oral pathogens

**DOI:** 10.3389/fimmu.2024.1349067

**Published:** 2024-03-01

**Authors:** Fernando García-Arévalo, Ana Gabriela Leija-Montoya, Javier González-Ramírez, Mario Isiordia-Espinoza, Idanya Serafín-Higuera, Dulce Martha Fuchen-Ramos, J. Gustavo Vazquez-Jimenez, Nicolas Serafín-Higuera

**Affiliations:** ^1^ Laboratorio de Biología Celular, Centro de Ciencias de la Salud Mexicali, Facultad de Odontología Mexicali, Universidad Autónoma de Baja California, Mexicali, BC, Mexico; ^2^ Facultad de Medicina Mexicali, Universidad Autónoma de Baja California, Mexicali, BC, Mexico; ^3^ Laboratorio de Biología Molecular, Centro de Ciencias de la Salud Mexicali, Facultad de Enfermería Mexicali, Universidad Autónoma de Baja California, Mexicali, BC, Mexico; ^4^ Instituto de Investigación en Ciencias Médicas, Departamento de Clínicas, División de Ciencias Biomédicas, Centro Universitario de los Altos, Universidad de Guadalajara, Tepatitlán de Morelos, Jal, Mexico; ^5^ Laboratorio de Microbiología, Facultad de Medicina, Universidad Autónoma de Baja California, Tijuana, BC, Mexico

**Keywords:** oral diseases, myeloid-derived suppressor cells (MDSCs), oral bacteria and fungi, inflammation, oral pathogens

## Abstract

The oral cavity presents a diverse microbiota in a dynamic balance with the host. Disruption of the microbial community can promote dysregulation of local immune response which could generate oral diseases. Additionally, alterations in host immune system can result in inflammatory disorders. Different microorganisms have been associated with establishment and progression of the oral diseases. Oral cavity pathogens/diseases can modulate components of the inflammatory response. Myeloid-derived suppressor cells (MDSCs) own immunoregulatory functions and have been involved in different inflammatory conditions such as infectious processes, autoimmune diseases, and cancer. The aim of this review is to provide a comprehensive overview of generation, phenotypes, and biological functions of the MDSCs in oral inflammatory diseases. Also, it is addressed the biological aspects of MDSCs in presence of major oral pathogens. MDSCs have been mainly analyzed in periodontal disease and Sjögren’s syndrome and could be involved in the outcome of these diseases. Studies including the participation of MDSCs in other important oral diseases are very scarce. Major oral bacterial and fungal pathogens can modulate expansion, subpopulations, recruitment, metabolism, immunosuppressive activity and osteoclastogenic potential of MDSCs. Moreover, MDSC plasticity is exhibited in presence of oral inflammatory diseases/oral pathogens and appears to be relevant in the disease progression and potentially useful in the searching of possible treatments. Further analyses of MDSCs in oral cavity context could allow to understand the contribution of these cells in the fine-tuned balance between host immune system and microorganism of the oral biofilm, as well as their involvement in the development of oral diseases when this balance is altered.

## 1 Introduction

Good oral health is essential for vital functions like eating, breathing, and speaking, and contributes to get confidence in interacting with others. Oral health is challenged by a range of diseases and conditions, with high prevalence and morbidity. Oral diseases such dental caries, periodontal disease, oral candidiasis, and dental pulp disease, are the most common noncommunicable diseases, affecting globally an estimated 3.5 billion people. These oral pathologies are increasing, particularly in low- and middle-income countries (1).

The estimated global average prevalence of caries of permanent teeth is 29%, and case numbers reach more than 2 billion cases. Untreated caries has many negative impacts in different phases of life, presenting pain and sleeping difficulties reduce quality of life and productivity (2). Irreversible pulpitis and pulp necrosis, without treatment, progress to apical periodontitis, an inflammation of the periapical periodontium, and commonly showing periapical bone resorption. The global prevalence of dental pulp pathology is about 52% (3). Periodontal disease has a global prevalence of about 19% in people aged greater than 15 years, representing more than 1 billion cases worldwide (2). Prevalence of periodontal disease starts in late adolescence, peaks around 55 years of age and remains high until old age. In its severe form, periodontitis is the sixth most prevalent condition in the world, and it affects about 10% of the adult population. Untreated periodontitis leads to progressive destruction of the tooth-attachment apparatus and eventual tooth loss, affecting oral functions and quality of life (4). The prevalence of peri-implantitis has been reported to be 56%, and the reported prevalence widely ranges from 12% and 43%. This difference in reported prevalence is due to a strict diagnostic criteria and case definitions of the disease. Peri-implantitis occurs in the hard and soft tissues surrounding dental implants. It results in adverse biological effects, such as bleeding on probing, suppuration, progressive bone loss and eventually implant removal (5). Oral candidiasis can occur in immunocompetent or immunocompromised patients, and it is the most common human fungal infection. More than 90% of patients with HIV develop oral candidiasis at some point of the course of disease. Greatest attention has been given to *Candida albicans* due to its prevalence in 60 to 90%. However, other species may be present, although less frequently (6). Sjögren’ syndrome is a systemic chronic autoimmune characterized by salivary and lacrimal glands immune-mediated damage, leading to dryness of the mouth and eyes. It was suggested that women and those in older age groups have the highest incidence and prevalence of this syndrome (7).

These oral diseases share a specific factor, which is the inflammatory condition, thus modulation of the oral inflammatory response must be critically precise to avoid the destruction of the affected oral tissues or, on the contrary, to prevent susceptibility to infections. Different inflammatory cells exhibit immunomodulatory capacity, such as Myeloid derived suppressor cells (MDSCs), which have been involved in oral diseases.

MDSCs represent a heterogeneous population of myeloid progenitor cells and immature myeloid cells derived from hematopoietic precursor, that are characterized for their immunosuppressive properties (8). MDSCs accumulate under pathological conditions and their immune suppressive activity responds in chronic inflammatory conditions such as infectious diseases, dysbiosis, autoimmune disorders, sepsis, trauma, and many other pathological conditions including cancer (8–10).

Even MDSCs involve a large range of phenotypes, two subpopulations of cells are identified in mouse and human according to their phenotypic and morphological similarity: monocytic (M)-MDSCs, defined in mouse as CD11b^+^Ly6C^high^Ly6G^–^ cells, and polymorphonuclear (PMN)-MDSCs or granulocytic (G)-MDSCs, which are defined as CD11b^+^Ly6C^−/low^Ly6G^+^ cells (11). Instead, in human M-MDSCs are defined as CD11b^+^CD14^+^CD33^+^HLA-DR^low/-^, and G-MDSCs as CD11b^+^ CD15^+^HLA-DR^low^CD66b^+^ (12). Other subpopulation of early-stage (e)-MDSCs was identified as HLA-DR^-^CD33^+^Lin^-^, in human (11, 13) and CD11b^+^Gr-1^−^F4/80^−^MHC-II^−^in mice (14). As shown, human MDSCs have been characterized by the expression of the common myeloid markers CD33 or CD11b, as well as the lack of mature myeloid cell markers such as HLA-DR. However, human MDSCs are far more complex and diverse phenotypes have been described in different tumors and infectious diseases (11).

MDSCs possess immunoregulatory functions that could prevent uncontrolled immune responses in situations requiring tolerance. However, in pathological situations of constant tissue damage and chronic inflammation, MDSCs could exacerbate tissue injury and promote inflammation (15). To achieve their immunosuppressive activity, the MDSC can stimulate the *de novo* generation of regulatory T cells and suppress T cells function through direct ligand-receptor engagement, release of soluble inhibitory cytokines, production of reactive oxygen species (ROS) and reactive nitrogen species (RNS) or depletion of amino acids essential for T cell (10, 16, 17).

In this sense, MDSCs stimulate the *de novo* generation of regulatory T cells through a process mediated by interferon (IFN)-gamma and interleukin (IL)-10. Furthermore, MDSCs can exert immunosuppressive effects on effector T cells by producing transforming growth factor (TGF)-β and IL-10. To contribute to the T cell elimination, MDSCs secrete ROS such as: superoxide anions, hydroxyl radicals, hydrogen peroxide, singlet oxygen and superoxide radicals produced by Nicotinamide adenine dinucleotide phosphate (NADPH) oxidase (NOX)1, NOX2, NOX3, and NOX4. Also, MDSCs can promote T cells apoptosis, through the activation of their inducible NO synthase (iNOS) resulting in the production of RNS: primarily nitric oxide (NO), that reacts with superoxide to produce peroxynitrites which promote the apoptosis of T cells and the nitration of the T-cell receptor (TCR), thus inhibiting T cell activation (10, 16). Depletion of amino acids as L- arginine or cysteine, through their conversion to different metabolites or by an increased uptake, are mechanism used by MDSCs to impair T cell functions. For example: in MDSCs, iNOS participates in L-arginine depletion by catalyzing the conversion of L-arginine to NO and L-citrulline. Also, arginase-1 is highly expressed by MDSCs and converts L-arginine to L-ornithine and urea. Moreover, MDSCs can increase the uptake of L-arginine by the CAT-2B transporter. As a result of these mechanisms, depletion of L-arginine leads to blockage of T cell proliferation and reduced TCR ζ-chain expression. On the other hand, cysteine is an essential amino acid for glutathione and DNA synthesis by T cells, and MDSCs can deplete cysteine by increasing the uptake through the SLC7A11 transporter, impairing ROS resistance and T cell activation (10, 16).

The contribution of MDSCs in oral cancer has been recently addressed (18–20) and it will not be revised. This narrative review focuses on the involvement of MDSCs in oral inflammatory diseases, as well as the relationship between MDSCs and major bacterial and fungal oral pathogens.

## 2 Myeloid-derived suppressor cells in oral inflammatory diseases

**Figure 1:**
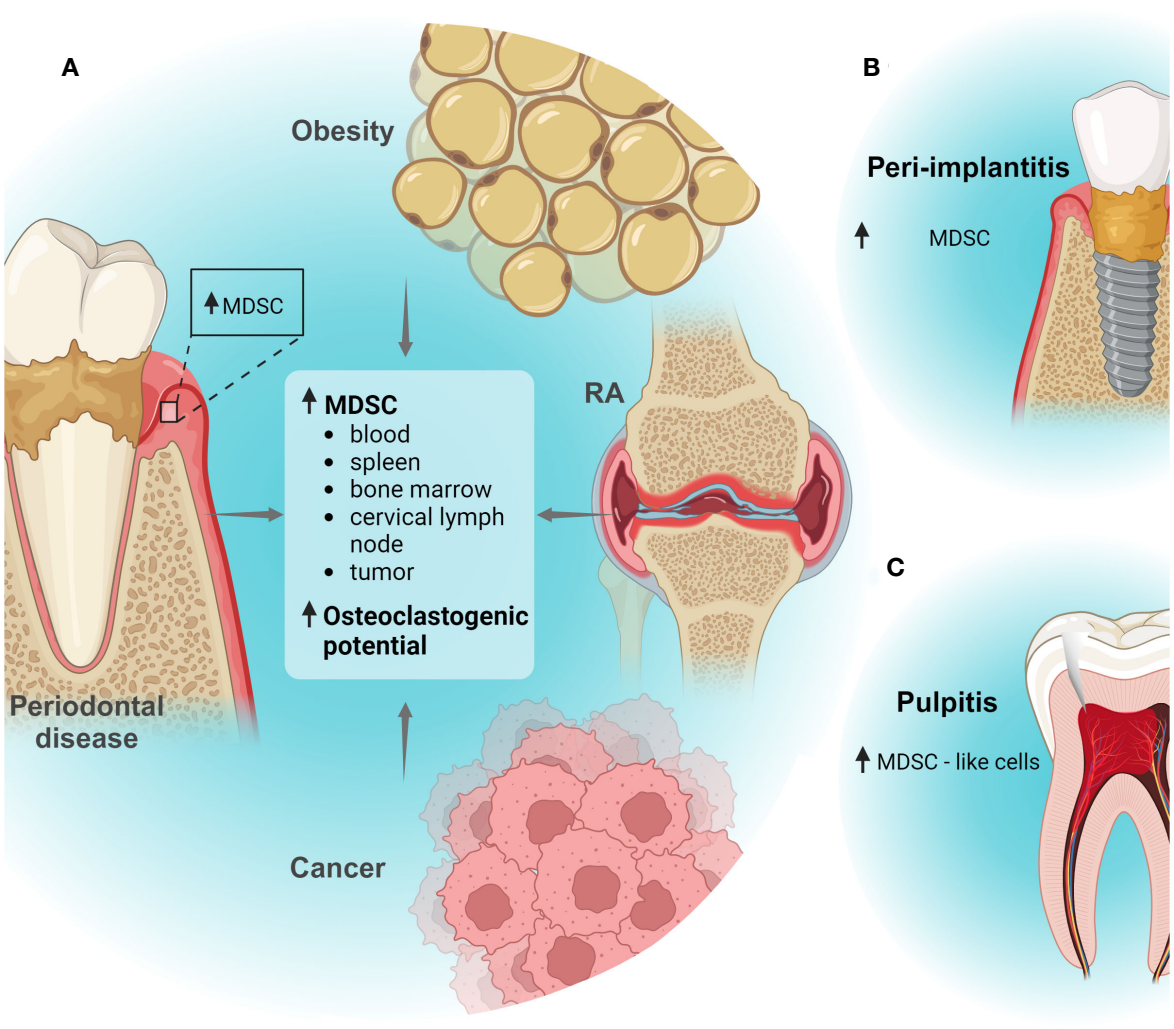
MDSCs in oral inflammatory diseases. **(A)** *In silico* analyses suggested that MDSCs are increased in human periodontitis tissues. Moreover, periodontal disease associated with other inflammatory conditions as obesity, rheumatoid arthritis (RA) or cancer can induce systemic expansion of MDSC subpopulations or recruitment to tissues. Also, periodontal disease and obesity can promote osteoclastogenic capacity of MDSCs. **(B)** *In silico* analyses suggested that MDSCs could increase in peri-implantitis tissues; **(C)** Increased infiltration of MDSC-like cells was detected dental pulp tissues in a pulpitis model. Created with BioRender.com.

The participation of MDSCs in different oral diseases has been analyzed in distinct reports (**Figure 1**). However, most studies have focused on certain diseases as periodontal disease and Sjögren’s syndrome. There is little or even no information on the contribution of MDSCs in most oral diseases.

### 2.1 Periodontal disease

Periodontal disease is largely caused by bacterial infections being one of the most common inflammatory disorders in humans. Periodontal disease comprises principally two illnesses, the gingivitis, a form of the disease involving inflammation of gingival tissue, and periodontitis, a condition encompassing damage of soft tissue and the continuing destruction of periodontal ligament and alveolar bone. Periodontitis has been widely linked to different inflammatory diseases as cardiovascular disease, type 2 diabetes, rheumatoid arthritis, inflammatory bowel disease, Alzheimer’s disease, and cancer. It was suggested que periodontitis can worsen these diseases through different mechanisms such as systemic dissemination of periodontal bacteria/bacterial components or through the oropharyngeal and orodigestive tract and release of inflammatory molecules to the blood, which could result in more inflammation (21, 22).

Previously, it was suggested that MDSCs could be involved in periodontal diseases (23) and since then different studies have analyzed MDSC in the context of this disease. For instance, bioinformatic analyses using the single-cell RNA-sequencing data of gingival tissues from healthy individuals and periodontitis patients suggested increased presence of M-MDSCs in periodontitis (24). Also, other independent bioinformatic studies analyzing whole-genome expression data of gingival tissues from healthy individuals and periodontitis patients suggested the increased presence of MDSCs in periodontitis tissues (25, 26). Together, these in silico analyzes suggest possible increased infiltration of MDSCs in inflamed gingival tissues. To the best of our knowledge, these are the first studies exploring the presence of MDSCs in gingival tissue from patients with periodontitis. Further reports must determine experimentally the expansion, suppressive functions, and subpopulations of MDSCs in patients with periodontitis. Moreover, future studies should stablish the role of MDSCs in human periodontal disease.

On the other hand, different studies have analyzed the participation of MDSCs in periodontal disease in association with other diseases using mice models (22). Diet-induced obesity mice generated by feeding high-fat diet showed increased expansion of MDSCs in spleen and blood (27). Additionally, the generation of experimental periodontitis in these mice, by a ligature tied in second molar, promotes more expansion of MDSCs in bone marrow and spleen. Even though, obesity can induce MDSC expansion, periodontitis can contribute to exacerbate this condition (27). Interestingly, experimental periodontitis can induce M-MDSC accumulation in spleen and bone marrow in mice fed with low-fat diet as compared with mice fed with low-fat diet without experimental periodontitis (27). Thus, it is possible that periodontitis could induce systemic expansion of MDSCs itself. Moreover, it was suggested that M-MDSCs induced by obesity could worsen the outcome of experimental periodontitis increasing alveolar bone lost (27, 28). In this sense, it was proposed that high-fat diet can generate M-MDSC reprogramming that resulted in increased osteoclastogenic potential (27, 28).

In other experiments where periodontitis was induced in mice by inoculation oral with *Porphyromonas gingivalis* bacteria, these mice did not show a significative increase of MDSCs in spleen and blood as compared with control mice (29). Interestingly, mice with *P. gingivalis*-induced periodontitis and rheumatoid arthritis showed expansion of M-MDSCs blood and more severe symptoms of arthritis compared with mice with rheumatoid arthritis and without periodontitis (29). Thus, periodontal disease and other inflammatory diseases can synergize to promote systemic expansion of MDSCs. It is possible that secretion of inflammatory molecules by both diseases explains the accumulation of MDSCs, however this remains to be determined.

Since periodontal disease has been linked to cancer development, gingival inflammation was generated in a mice model of early breast cancer metastasis by the injection of LPS in gingiva and breast cancer cell line 4T1 injection in the mammary fat pad (30). The presence of periodontal disease promoted more micrometastasis and infiltration of MDSCs in cervical lymph nodes. It was suggested that secretion of IL-1β and expression of C-C Motif Chemokine Ligand (CCL)2, C-X-C Motif Chemokine Ligand (CXCL)12, CCL5, and CXCL5 by cells such as fibroblast in the inflamed gingiva induced the recruitment of MDSCs. Moreover, in a mice model of late breast cancer metastasis, the gingival inflammation generated more MDSC infiltration in cervical lymph nodes and head and neck tissues (30). Thus, it is possible that periodontal disease induces generation of inflammatory molecules with potential capacity to recruit MDSCs.

Together, these studies suggest that periodontal disease can promote systemic expansion of MDSC subpopulations in association with other diseases (**Figure 1A**). Importantly, MDSC biological functions could represent a bidirectional mechanism that worsens/modulate the outcome of some diseases with a significant inflammatory component that occur simultaneously in an individual.

### 2.2 Sjögren’s syndrome

Sjögren’s syndrome is a chronic autoimmune disorder affecting secretory glands, with inflammatory cell infiltration of the salivary and lacrimal glands, causing xerostomia (dry mouth) and xerophthalmia (dry eye) by the acinar epithelial cell atrophy, cell death, and loss of exocrine function. The etiology of this disease remains unclear. Sjögren’s syndrome can be primary (when it presents alone) and secondary (when it presents in association with other autoimmune diseases) (31–33). Different mice models to study pathogenesis of Sjögren’s syndrome have been reported, which partially exhibit immunological and clinical characteristics of this disease (33). Mice models can be genetic (animals develop disease symptoms spontaneously due to genetic mutations or modification) or induced (the disease is artificially induced) (33).

The nonobese diabetic (NOD) mouse model is a strain that develops Sjögren’s Syndrome-like disease with infiltration of inflammatory cells in the exocrine glands and affected secretion of saliva and tears, as well as the presence of serum autoantibodies associated to this disease (33). Since NOD mice develop both autoimmune diabetes and Sjögren’s Syndrome-like disease can be considered a model of Secondary Sjögren’s syndrome (33). An advantage of this model is that the disease develops spontaneously; additionally, the model has allowed the analysis of different immune factors in the development of the disease (33). MDSCs have been analyzed in this mice model. 4-week-old NOD mice with absence of Sjögren’s Syndrome-like symptoms showed decreased percent of MDSCs (CD11b^+^Gr-1^+^) in blood compared with 8, 10, 12-week-old NOD mice, which showed progressive infiltration of T-cells in submandibular glands and gradual reduction in salivary flow rate (features that resemble Sjögren’s syndrome) (34), as well as increased levels of serum lL-12 (35). Additionally, other study reported the increased expansion of MDSCs in spleen, lymph node and bone marrow of 14-week-old NOD mice compared with 6-week-old NOD mice (with absence of Sjögren’s Syndrome-like symptoms) (36). Thus, MDSC expansion was accentuated when the severity of the disease increased. The PMN-MDSC (CD11b^+^Ly6G^+^Ly6C^low^) subpopulation presents more expansion in blood and bone marrow of 12-week-old NOD mice. In contrast, M-MDSC (CD11b^+^Ly6G^-^Ly6C^high^) subpopulation were decreased (37). Curiously, infiltrated CD11b^+^Gr1^+^ cells have been detected in submandibular glands of mice; however, this population is not increased in NOD mice showing Sjögren’s Syndrome-like symptom (34). Therefore, the effect of MDSCs on Sjögren’s syndrome-like symptoms, such as reduced salivary flow, might result from a primarily systemic effect rather than a local effect.

Multimolecular complexes created to participate in defense against infectious agents, called inflammasomes, can induce maturation and release of IL-1β and IL-18. Inflammasomes and these interleukins are aberrantly expressed and activated in NOD mice with Sjögren’s Syndrome-like manifestation (38). Interestingly, the NLR Family Pyrin Domain Containing 3 (NLRP3) inflammasome is highly expressed and activated in splenic MDSCs of NOD mice with Sjögren’s Syndrome-like manifestation; thus, these cells produce increased levels of IL-1β. Treatment with LPS of NOD mice aggravated the Sjögren’s Syndrome-like manifestations, induced expansion of MDSCs, promoted the activation of (NLRP3) inflammasome and production of IL-1β and IL-18 (38). Moreover, the LPS treatment increased expression and activation of NLRP3 in MDSCs. While the treatment with an inhibitor of NLRP3 generated the contrary effects. Additionally, it was suggested that activation of NLRP3 in MDSCs of NOD mice is induced by IL-27, a cytokine showing pro-inflammatory and anti-inflammatory effects with roles do not elucidated completely in Sjögren’s Syndrome (38). Thus, this study suggests that inflammasomes could represent possible therapeutic targets.

It was proposed that MDSCs from NOD mice with Sjögren’s Syndrome-like symptom express high levels of Fc gamma receptor (FcγR)IV, which can bind Fc fragments present in immune complex and modulate inflammatory response. Also, MDSCs showed changes in the expression of glycolysis-associated genes, and this was associated to potential pro-inflammatory and changes of the T cell populations (36). Moreover, MDSCs promoted inflammation in the NOD model and inhibit the Th2 cell response as well as production of serum IL-4 (34). In accordance with the observations described in the NOD mice, it was reported that HLA-DR^-^CD11b^+^CD33^+^ MDSC population is increased in blood of patients with Sjögren’s syndrome, and this correlated with the reduction of serum IL-4 in these patients (34). Moreover, the expansion of MDSCs in Sjögren’s syndrome patients correlated positively with the increase of Th17 cells in blood, high levels of serum autoantibodies, IgG levels and systemic disease activity (36). The antibody deposition with cross-reactive against autoantigens can form immune complex, and the treatment of MDSCs with heat-aggregated IgG, simulating immune complex, promoted the alteration in the balances of the T cells populations increasing ratio of Th17/Treg cells and decreasing the ratio of Th1/Th2; as well as changes in the metabolism of glycolysis in MDSCs with increased expression of glycolysis-associated gene, such as glucose transporter 1 (Glut1), hexokinase 2 (HK2) and lactate dehydrogenase A (LDHA). Similarly, MDSCs from patients disrupted the balances of T cells populations and showed increased glucose uptake capacity, expression of Glut1, HK2, LDHA, and transcription factor hypoxia inducible factor 1 subunit Alpha (HIF-1α) and mTOR phosphorylation, *in vitro* (36). Mechanistically, it was observed that MDSCs from Sjögren’s syndrome patients express highly FcγRIII and MDSC FcγRIII^+^ population is expanded in their blood. Fc gamma receptor binds to the Fc fragments of IgG of immune complexes which activates immunoreceptor tyrosine-based activation motif (ITAM), then this promotes the activation of mTOR and expression of HIF-1α, resulting in increased glycolysis and modulation of the observed proinflammatory effects (36).

Also, MDSCs have been analyzed in a model of primary Sjögren’s syndrome (39). In this induced model, mice are immunized with salivary gland homogenate emulsified in Freund’s incomplete adjuvant to develop experimental Sjögren’s syndrome. CD11b^+^Gr-1^+^ MDSC cells were increased in spleen, blood, cervical lymph nodes and salivary glands of these mice; similarly, CD11b^+^Ly6G^-^Ly6C^high^ (M-MDSCs) and CD11b^+^Ly6G^+^Ly6C^low^ (PMN-MDSCs) populations were expanded in spleens (39). Interestingly, splenic MDSCs of mice in early stage of experimental Sjögren’s syndrome (10 days postimmunization) showed an immature phenotype (low expression levels of CD40, CD80, CD86 and MHC-II markers) and potent suppressive activity of T cell proliferation. While MDSCs of late stage of experimental Sjögren’s syndrome (10 weeks postimmunization) expressed markers of more differentiated mature phenotype (high expression levels of CD40, CD80, CD86 and MHC-II markers), drastic reduction of suppressive activity and decreased arginase activity and NO levels. Adoptive transference of early stage MDSCs in the induced model reduces the severity of features that resemble Sjögren’s syndrome, improving the saliva flow rate to normal level, decreasing the lymphocytic infiltration in salivary glands, and reducing the levels of serum autoantibodies against antigens of salivary glands. Moreover, the early stage MDSCs reduce the Th1 and Th17 responses. On the other hand, the adoptive transference of late stage MDSCs did not show changes or improvements in the analyzed characteristic of the syndrome (39). Thus, MDSC immunosuppressive potential affects to the development of the disease.

It was suggested that changes in the suppressive activity and level of MDSC immaturity observed in the experimental Sjögren’s syndrome model can be modulate by the proteins glucocorticoid-induced TNFR family–related protein (GITR) and the GITR ligand (GITRL). GITR is expressed by MDSCs and treatment with GITRL promotes expression of markers of differentiated and mature phenotype, reduces MDSC suppressive activity of T cell proliferation and decreases arginase activity and levels of NO in MDSCs. Increased expression of GITRL is observed in the spleen and salivary glands during disease progression in the experimental model of Sjögren’s syndrome (39). Also, the GITR expression in MDSCs is gradually increased with the progression of the disease. In accordance, patients with primary Sjögren’s syndrome present increased levels of serum GITRL with expansion of MDSCs which express high levels of GITR and produce low levels of Arginase. Thus, GITR/GITRL pathway modulate function of MDSCs in Sjögren’s syndrome (39). The proposed molecular mechanism involved the bind of GITRL to GITR present in membrane of MDSCs, which induces the recruitment of the adaptor protein TNF receptor associated factor (TRAF)3 and the attachment of the Protein Phosphatase 2A (PP2A). Then, PP2A dephosphorylates to phosphatase and tensin homolog (PTEN) at Ser380 region. This decreases phosphorylation levels of Serine/Threonine Kinase (AKT) and reduces its activity, resulting in diminished levels of phosphorylation of signal transducer and activator of transcription (STAT)3 and subsequent affectation in MDSC immunosuppressive capacity in late stage of experimental Sjögren’s syndrome (39).

Taken together, these observations suggest that even though, the expansion of MDSCs affects the progression of the Sjögren Syndrome, the phenotype and potential suppressive of MDSCs are important modifiers in the result of the disease. The generation, characteristics, and functions of MDSCs could fluctuate in response to signals in the inflammatory microenvironment; the plasticity can be useful in the establishment of potential treatments. Therefore, possible treatments should impact in the qualitative and quantitative aspects of MDSCs.

#### 2.2.1 Experimental treatments targeting MDSCs

It was suggested that IL-12 notably promotes MDSC accumulation and expansion in bone marrow and spleen of NOD mice resulting in worsening of features that resemble Sjögren’s syndrome. The importance of IL-12 in the MDSC expansion is depicted in mice deficient in IL-12 that show decreased percentage of MDSCs in spleen. Interestingly, treatment of NOD mice with anti-IL-12 relieves Sjögren’s syndrome-like symptoms and reduce MDSC expansion (34, 35). On the other hand, adoptive transference of MDSCs to NOD mice aggravates the features resembling Sjögren’s syndrome. However, reduction of the MDSC population in spleen and blood of NOD mice by Anti-Gr-1 treatment relieved the symptoms (34). In addition to treatments targeting MDSC expansion in NOD mice such as the use of Anti-Gr-1 or Anti-IL-12, transference of mesenchymal stem cells (MSCs) has been established in this model (37). These cells have been proved as experimental treatment in different autoimmune diseases. The transfer of human umbilical cord-derived MSCs to NOD mice with Sjögren’s syndrome-like symptoms reduced the expansion of the PMN-MDSC and M-MDSC subpopulations in blood and bone marrow and ameliorated symptoms of the disease. Moreover, MSCs promoted expression of arginase and INOS in MDSCs which could result in increased immunosuppressive activity. It was suggested that the production of cyclooxygenase (COX)2, prostaglandin (PG)E2 and IFN-β by MSCs prevented MDSC partial generation and induced the incremented immunosuppressive activity (37).

Also, treatments targeting MDSCs using the model of primary Sjögren’s syndrome have been reported. Adoptive transference of MSCs from mouse bone marrow to the experimental Sjögren’s syndrome mouse model amended the symptoms of the disease with reduced levels of serum autoantibodies against salivary glands antigens, recovery of the saliva flow rate and decreased lymphocytic infiltration in salivary glands; as well as diminished Th1 and Th17 response. In contrast with the observed with NOD mice treated with MSCs; the experimental Sjögren’s syndrome mouse model treated with MSC showed expansion of PMN-MDSCs and M-MDSCs in spleen and cervical lymph node (37, 40). This apparent discrepancy could be explained by differences in the analysed mice models and the tissue from which the MSCs were obtained. Interestingly, the generated MDSCs after of MSC treatment augmented levels of NO and arginase activity and exhibited increased immunosuppressive activity by inhibiting T cell proliferation. It was suggested that TGF-β secreted by MSC induce the phosphorylation of Smad2/3 in MDSCs inducing an immature state and increased suppressive function of MDSCs and resulting in relief from the symptoms (40). Importantly, these observations were consistent with results reported in NOD mice treated with MSCs. This observation is important because it suggest that MSCs amended the symptoms of the disease in part modifying the phenotype of MDSCs. Additionally, MSCs from bone marrow or from the olfactory lamina of mice can secrete vesicles of 50-150 nm expressing CD9 and CD63. Treatments with these vesicles or with MDSCs treated with the vesicles, using the experimental Sjögren’s syndrome mouse model, generated similar effects to the adoptive transference of MSCs (41). Analyses in the induced mice model and *in vitro* showed that MSCs-secreted vesicles can be internalized by MDSCs resulting in more expansion, maintenance of immature state, increased immunosuppressive activity with augmented levels of NO, ROS and arginase activity (41). Mechanistically, it was suggested that the observed effects could be due to MSCs-secreted vesicles contain IL-6 and S100A4 protein; IL-6 is delivered to MDSCs activating Jak2/Stat3 pathway. On the other hand, S100A4 binds to TLR4 in MDSCs, which promotes the production of IL-6 by MDSCs generating the effects in an autocrine way (41). Together, these observations suggest that MSCs can modulate suppressive function, phenotype, and expansion of MDSCs by secretion of different cytokines and secretion of vesicles.

Also, MDSCs can produce vesicles that can produce immunomodulatory effects. Splenic MDSCs from tumor-bearing mice, which are greatly immunosuppressive, can produce vesicles of 50-150 nm expressing CD9, CD63 and TSG101. Treatments with these vesicles using the experimental Sjögren’s syndrome mice ameliorated symptoms and attenuated the progression of the disease (42). Interestingly, the population of germinal center B cells (CD19^+^Fas^+^GL7^+^) was reduced in spleen and cervical lymph node. *In vitro* analyses showed that the vesicles inhibit proliferation and differentiation of germinal center B cells, which participate in progression of disease. It was proposed that the vesicles contain the microRNA miR−10a−5p, which bind 3´-UTR of the transcriptional repressor B cell lymphoma 6 (BCL6) mRNA, reducing its expression. Bcl6 is needed for the development and function of germinal center B cells (42, 43). Additionally, analyses of the specific profiles of cargo molecules into the vesicles and secretome could allow identify more therapeutic targets. Interestingly, Anti-Ly6G treatment reduced PMN-MDSC population in experimental Sjögren’s syndrome mice and resulted in the expansion of population of germinal center B cells, increased Th1 and Th17 responses and exacerbated disease progression (accelerated decrease in salivary flow rate and raised serum levels of autoantibodies) (44).. These showed the importance of PMN-MDSCs in disease development.

**Figure 2:**
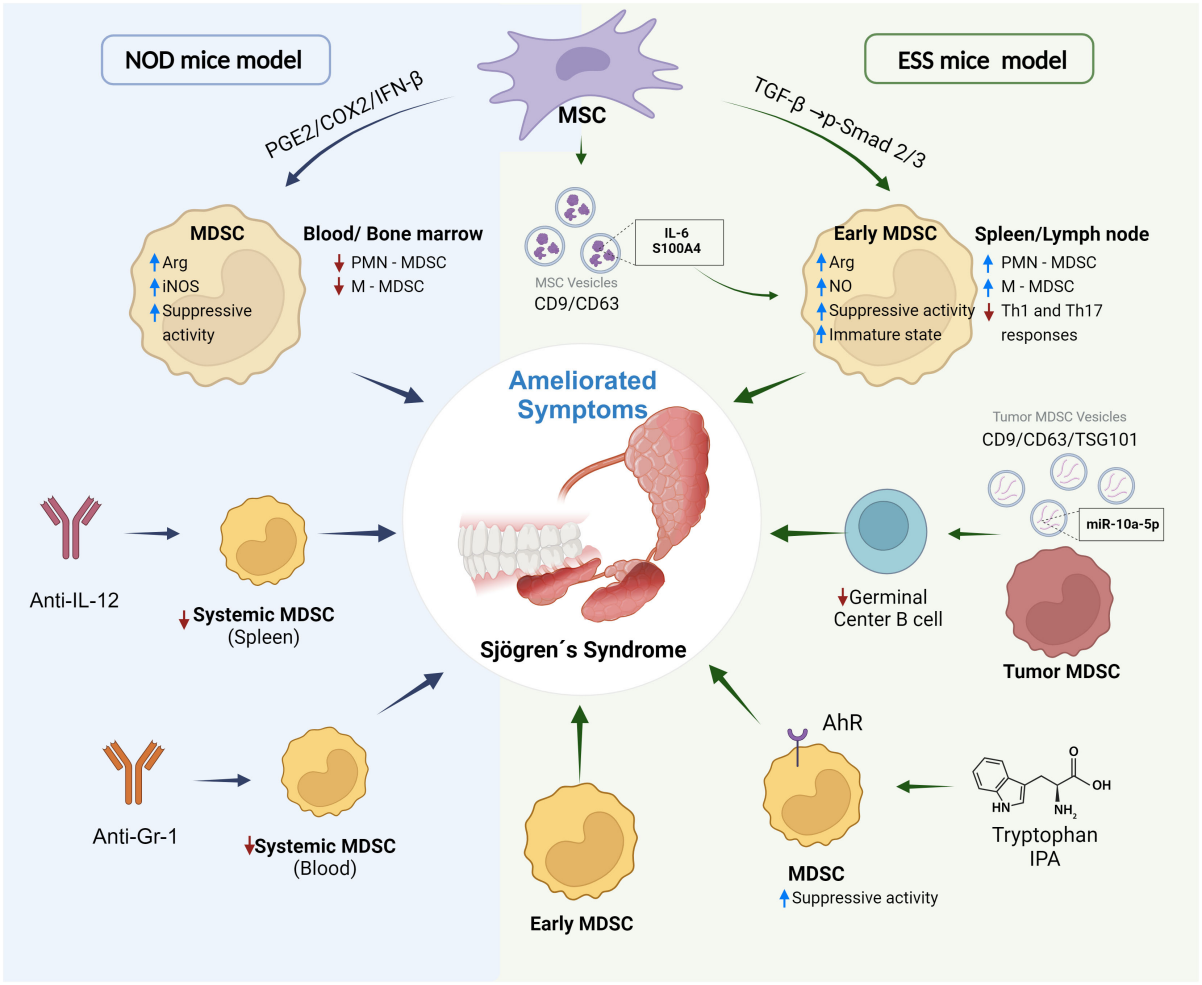
MDSCs as a target in experimental treatments of Sjögren’s syndrome. Different strategies have been reported to ameliorate symptoms and progression of Sjögren’s syndrome targeting MDSCs and using NOD mice and induced experimental Sjögren’s syndrome (ESS) mice model. Treatments with Anti-IL-12, Anti-Gr-1 or transference of MSC (mesenchymal stem cells) in NOD mice reduce expansion of MDSCs and increase of immunosuppressive activity by PGE2/COX2/IFN-β pathway. On the other hand, MSC can induce expansion of MDSCs with high immunosuppressive activity by TGF-β/Smad pathway or secretion of extracellular vesicles transporting and delivering inflammatory molecules. Immunosuppressive MDSC from tumors can reduce generation of germinal center B cells by production of extracellular vesicles delivering microRNA miR-10a-5p. IPA (Indole-3-propionic acid) and molecules derived from tryptophan metabolism can be ligands of the receptor AhR (aryl hydrocarbon receptor) expressed in MDSCs. The binding of the ligand promotes immunosuppressive activity and generation of MDSCs. Treatment with Early MDSCs [MDSCs of mice in early stage of experimental Sjögren’s syndrome that show an immature phenotype (low expression levels of CD40, CD80, CD86 and MHC-II markers) and potent suppressive activity of T cell proliferation] relieved symptoms of the disease. Created with BioRender.com.

The aryl hydrocarbon receptor (AhR) is a ligand-activated transcription factor that participates in modulation of the immune and inflammatory response (45) and it is expressed in human and mouse PMN-MDSCs (44). Indole-3-propionic acid (IPA), a AhR ligand derived from tryptophan metabolism, promoted more PMN-MDSC generation and increased MDSC immunosuppressive activity by increasing NADPH oxidase activity, resulting in augmented ROS production. Also, mice with a tryptophan-free diet showed reduced PMN-MDSC expansion and low levels ROS production by PMN-MDSCs (44). Thus, AhR signalling is important in the modulation of PMN-MDSC functions. PMN-MDSCs from NOD mice and mice model of primary Sjögren’s syndrome expressed low levels of AhR in consistence with reduced immunosuppressive capacity and impaired ability to produce ROS (44). It was suggested that the transcription factor, the interferon regulatory factor 4 (IRF4) could bind the promoter of AhR and inhibit its expression. IRF4 is overexpressed with disease progression in the primary Sjögren’s syndrome model. Moreover, AhR antagonist treatments or tryptophan-free diet in mice model of primary Sjögren’s syndrome showed similar effects to that observed with Anti-Ly6G treatment exacerbating development of the disease (44). This suggested that AhR signalling in PMN-MDSCs can modulate progression of the disease. Interestingly, IPA treatments in the mice model diminished progression of the disease in concordance with increased immunosuppressive activity and expansion of PMN-MDSCs (44). Together these observations suggest that MDSCs contribute widely to the development and progression of Sjögren’s syndrome, and they could be a possible therapeutic target (**Figure 2**).

### 2.3 Other oral diseases involving MDSCs

#### 2.3.1 Peri-implantitis

Another infectious disease is the peri-implantitis; in this inflammatory condition associated to oral microbial dysbiosis, the soft and hard tissues surrounding dental implants are affected (46). Previously, a bioinformatic analysis examined groups of genes that are representative for immune cell subpopulations and suggested that MDSCs could be involved in the inflammatory processes that take place during peri-implantitis (47) (**Figure 1B**). However, experimental detection of MDSCs in peri-implant tissues or functional analyses as immunomodulatory activity or osteoclastogenic potential of these cells in peri-implantitis have not been performed.

#### 2.3.2 Pulpitis

The dental pulp is a specialized tissue and highly innervated that allow to maintain the biological and physiological vitality of the tooth. Connective tissue, nerve fibers, blood vessels and diverse cells conform the pulp (48). Pulpitis, an inflammatory response of the dental pulp, can be triggered by mechanical, chemical, and microbiological factors; however, bacterial infection is the most common cause of pulpal inflammation (49). Previously, in an experimental pulpitis model, using rat incisor dental pulp tissue treated with LPS, MDSC-like cells were detected by flow cytometry using the markers CD45^+^TCRαβ^-^CD161^Low^ (50) (**Figure 1C**). However, immunosuppressive activity of these cells has not been determined yet. Interestingly, markers to analyse rat MDSCs or to distinguish PMN-MDSCs and M-MDSCs from rat are limited. The markers CD11b/c and His48 has been more reported, however markers as CD161 and CD172a also have been utilized (51). Additional studios including more MDSC markers, functional analyses and diverse models of pulpitis could determine the contribution of MDSCs in the inflammatory microenvironment during this disease.

## 3 Myeloid-derived suppressor cells and oral bacteria

### 3.1 Fusobacterium nucleatum

*F. nucleatum* is a Gram- negative anaerobic bacterium, member of the human oral microbiome of both healthy and diseased individuals, it is commonly present in the urogenital tract, intestinal tract and upper digestive tract. As an oral opportunistic pathogen, *F. nucleatum* plays an important role in the transition from healthy to diseased state. Thus, it is involved in the development of periodontal disease, peri-implant diseases, respiratory diseases and recently, it has been associated to oral and colorectal carcinogenesis (52–54). In host healthy conditions, *F. nucleatum* stimulates the production of antimicrobial peptides, chemokines, and cytokines by the epithelial cells to avoid incurring infections and preserve the oral health (53). Otherwise*, F. nucleatum* is an intermediate colonizer of the oral biofilm, and due its multiple adhesins, has an important role in the oral biofilm formation, acting as a bridge between initial aerobic colonizer and the late, highly virulent, anaerobic colonizers, thereby promoting inflammation and disease progression (53). *F. nucleatum* also mediate invasion and facilitating the spread of bacteria, it became systemic due to its ability to damage the epithelial barrier, stimulate the loss of intercellular adhesion in the endothelium, and increase vascular permeability (53, 55). Given its ability to stimulate oncogenic pathways*, F. nucleatum* is now considered a cancer -leading (52).

Presence of *F. nucleatum* can induce MDSC generation efficiently *in vitro* (56) and infection with *F. nucleatum* per se can promote accumulation of MDSCs in colon of mice (57). However, the relationships between MDSCs and *F. nucleatum* in the context of oral diseases has not been evaluated. Knowledge of this relationship comes mainly from studies that analyze the presence of the bacteria in different types of cancer.

*Fusobacterium* species are found in pancreatic cancer tissues; moreover, *F. nucleatum* can colonize pancreatic tumors which have been associated to malignant potential and poor prognosis (58). Recently, it was proposed that *F. nucleatum* can attract and induce infiltration of MDSCs in pancreatic tumors. Mechanistically, it was suggested that intratumor *F. nucleatum* can induce the secretion of chemokines, such as CXCL1, by pancreatic cells promoting attraction of MDSCs expressing the C-X-C motif chemokine receptor (CXCR)2 (58). The infiltration of MDSCs and the promotion of inflammatory molecules by invasion of *F. nucleatum* could promote tumoral microenvironment (58).

Furthermore, peroral administration of *F. nucleatum* in a mice model of intestinal tumorigenesis promoted more infiltration of M-MDSCs and PMN-MDSCs in tumors (59). Also, peroral treatment with *F. nucleatum* in mouse model of colorectal cancer induced more accumulation of MDSCs in tumors. Remarkably, the reduction/elimination of *F. nucleatum* colonization by treatment with antibiotic cocktail or with a silver nanoparticle-linked M13 phage that specifically kills *F. nucleatum*, resulted in decreased infiltration of MDSCs in tumors of these mice (60). In this sense, *Fusobacterium* has been detected in samples of human colorectal cancer. It was suggested that mechanistically, *F. nucleatum* can induce accumulation of MDSCs in tumoral tissues through modulation of the function and increase of invariant natural killer T (iNKT) cell in tumors (61). iNKT cells are a glycolipid-specific population of T cells that produce rapid and early cytokine responses and include different subsets with a particular transcriptional regulation and a distinctive profile of secreted cytokines (62). *F. nucletaum* promotes pro-tumorigenic phenotype of iNKT cells, which can produce chemokines of the C-X-C and C-C motif ligand family genes CXCL8, CXCL2, CXCL3, CCL3L1, CCL4L2, CCL20, and CCL22, as well as colony stimulating factor 2 (GM-CSF) and IL-17 resulting in MDSC recruitment (61). Interestingly, iNKT cells has been involved in inflammation of oral tissues (63); however, the relationship between these cells and MDSCs in oral context have not been addressed.

**Figure 3:**
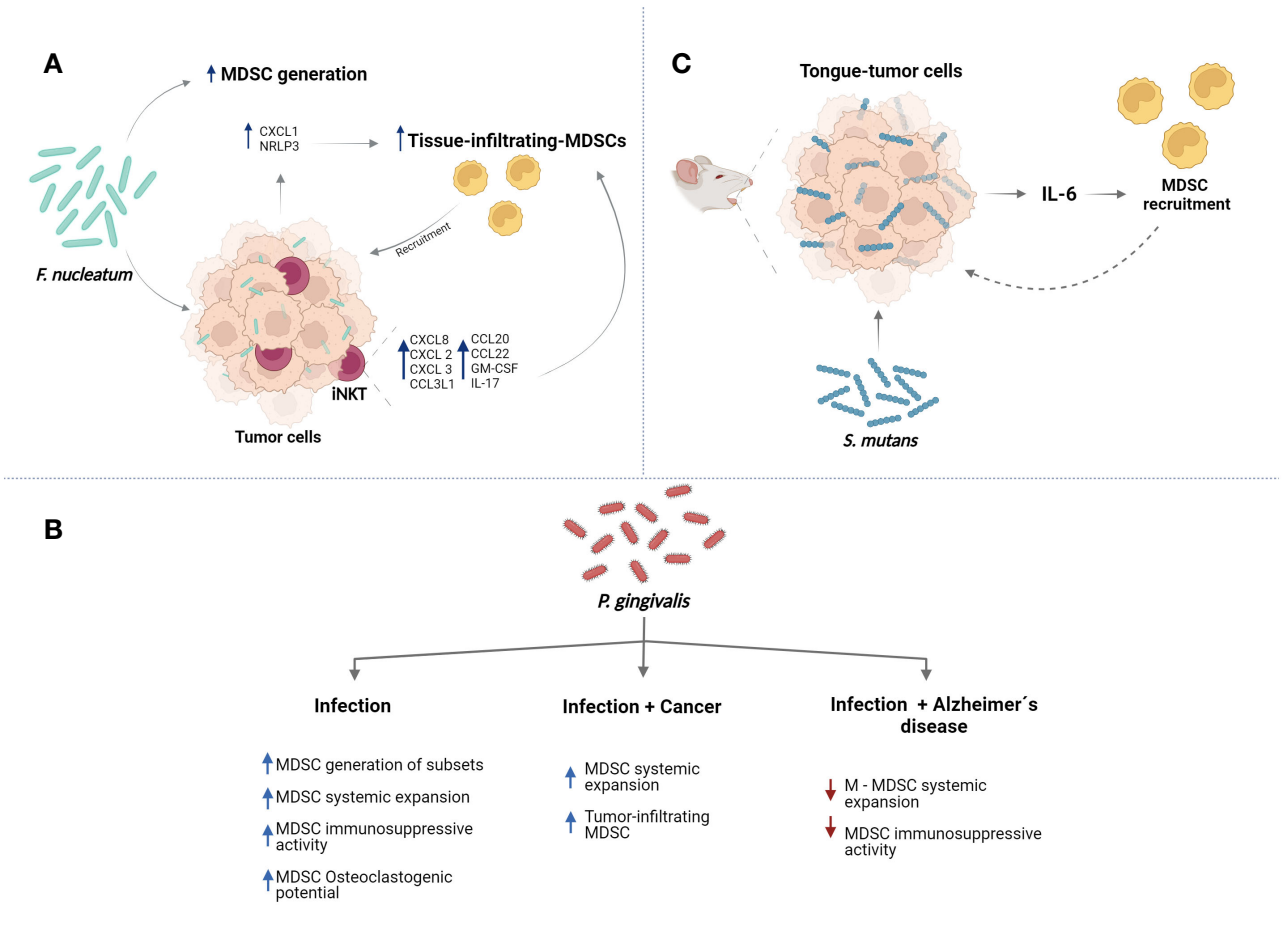
Major oral bacteria modulate the functions of MDSCs. **(A)** *F*. *nucleatum* can promote the generation of MDSCs. Also, the bacteria can induce the MDSC recruitment to tissues inducing expression of proinflammatory molecules such as CXCL1 and activation of NLRP3 in tumors cells. Additionally, *F*. *nucletaum* can promote recruitment of iNKT cells which can express proinflammatory molecules resulting in more tissue-infiltrating MDSCs. **(B)** *P. gingivalis* can modulate different functions of MDSCs in the context of infection, cancer, and Alzheimer´s diseases. **(C)** *S. mutans* can induce the expression of IL-6 by tumor cells promoting recruitment of MDSCs to tissues. Created with BioRender.com.

Presence of *F. nucleatum* in tissues of liver metastasis from patients presenting colorectal cancer has been associated with increased infiltration of MDSC-like cells in metastatic sites (64). In accordance, oral treatment with *F. nucleatum* promoted more accumulation of MDSCs in metastatic tissues of a mice model of liver metastasis. Increased levels of IFN-γ, TNF-α, IL-6, IL-12, IL-17A, chemokine CXCL1, IL-9, and macrophage chemoattractant protein-1 (MCP-1) in serum of these mice were observer (65). Also, increased infiltration of MDSC-like cells in esophageal squamous cell carcinoma tissues from patients was associated with the presence of *F. nucleatum* in the tumors. *In vitro* analyses showed that presence of *F. nucleatum* promotes expression of NLRP3 in the esophageal squamous cell carcinoma cells, which was necessary to induce efficiently generation of MDSCs and their recruitment (56). Together, these reports suggest that bacteria could modulate inflammatory profile in the different tissues of the cancer mice models resulting in the recruitment and expansion of MDSCs (**Figure 3A**). Modulation of MDSC biological functions by *F. nucleatum* in the different anatomical sites of the mouth in health or disease has not been determined.

### 3.2 Porphyromonas gingivalis

*P. gingivalis* is a Gram-negative anaerobic coccobacillus that mainly colonizes the periodontal pockets and is considered a keystone pathogen in the development of periodontal disease. The pathogenicity and survival strategies of *P. gingivalis* are mainly supported by different virulence factors such as: fimbriae, cysteine proteinases, hemagglutinins, heat shock proteins, gingipains, outer membrane vesicles, and lipopolysaccharide (LPS). These factors are involved in different mechanisms to improve adhesion and invasion capabilities, the induction of peripheral CD4+ T helper cells to produce proinflammatory cytokines, the activation of T lymphocyte immune response and (RANKL)-induced osteoclast, attenuation of dendritic cell-induced chemokine responses and deliver virulence factors (66–68). *P. gingivalis*, after colonizing subgingival tooth biofilms, gets access to gingival tissues and is strongly associated with the development and progression of periodontal diseases, severe periodontitis and peri-implantitis lesions, also *P. gingivalis* could be related to the etiology of oral, oropharyngeal, and esophageal cancer. Moreover, *P. gingivalis* can migrate from periodontal tissue to different organs as a result of traumatic activities by entering to the vasculature through ulcerated epithelium and lymph vessels. Thus, *P. gingivalis* has been detected in different organs and correlated with the occurrence and development of systemic diseases such as atherosclerosis, Alzheimer’s disease rheumatoid arthritis, diabetes and adverse pregnancy outcomes. Also, the contribution to respiratory disease, nonalcoholic fatty liver and depression have been reported (54, 69–72).

*In vitro*, *P. gingivalis* can induce more M-MDSC generation with different efficiency depending on the presence of *P. gingivalis* strains (29). Additionally, the biological effects of *P. gingivalis* on MDSCs have been analysed in different mouse models (recently revised in (22)). Treatment of mice with heat-killed *P. gingivalis* promoted increased accumulation of MDSCs in spleen and blood with capacity to induce downregulation of the TCR ζ-chain and to disrupt T cell function (73). Also, chronic systemic infection of mice with freshly harvested *P. gingivalis* induced more MDSC expansion in bone marrow and spleen with high immunosuppressive capacity (74). These MDSCs showed different phenotype (CD11b^+^Ly6G^high^Ly6C^int^, CD11b^+^Ly6G^int^Ly6C^high^, and CD11b^+^Ly6G^int^Ly6C^int^). Interestingly, the subpopulation CD11b^+^Ly6G^int^Ly6C^high^ showed more immunosuppressive activity compared with the other subpopulations and capacity to differentiate into osteoclasts in the presence of RANKL and M-CSF, which was promoted by *P. gingivalis*; suggesting that can be precursor of osteoclasts (74). More characterization showed that population CD11b^+^Ly6G^int^Ly6C^high^ MDSCs can express colony stimulating factor 1 receptor (c-fms) (receptor of M-CSF) (75); this M-MDSC subset called CD11b^+^c-fms^+^ increased in bone marrow and spleen of mice infected with *P. gingivalis* and showed immunosuppressive activity (75). Presence of *P. gingivalis*, M-CSF and RANKL promoted increased differentiation of CD11b^+^c-fms^+^ MDSCs into osteoclasts resulting in more bone resorptive activity. RANK^+^CD11b^+^c-fms^+^ or RANK^-^CD11b^+^c-fms^+^ MDSC subsets showed osteoclastogenic activity (75). In an independent report, *P. gingivalis* promoted systemic expansion of CD11b^+^c-fms^+^Ly6C^high^ MDSC cells with high capacity to differentiate to osteoclasts (76). This MDSC subset presents a transcriptional profile associated to osteoclasts with expression of Ctsk (Cathepsin K), MMP9 (matrix metallopeptidase-9), OC-associated receptor (Oscar), OC stimulatory transmembrane protein (OCstamp), and the calcium-binding proteins S100A8 and S100A9 (76).

Together, these reports suggest that *P. gingivalis* infection can promote expansion and generation of MDSCs. Also, the bacteria can modulate MDSC phenotypes and biological functions such as immunosuppression and osteoclastogenic activity. Interestingly, *P. gingivalis* is part of the oral biofilm; however, these biological effects on MDSCs by *P. gingivalis* has not been analysed in the diverse anatomical sites of mouth in where a complex equilibrium must be maintenance between oral biofilm and immune response of the host.

Chronic inflammation related with presence of *P. gingivalis* and immunosuppressive capacity of MDSCs have been associated to cancer. Thus, the infection with this bacterium and the biological effects on MDSCs have been analysed in the context of cancer (77, 78). Oral treatment with *P. gingivalis* in mice models of oral squamous cell carcinoma and esophageal squamous cell carcinoma can promote more malignant progression and recruitment of MDSCs (77, 78). It was suggested that *P. gingivalis* can colonize oral precancerous lesions and provoke that the keratinocytes express molecules such as CCL2, CXCL2, IL-6, and IL-8, which result in the MDSCs recruitment (78). Also, *P. gingivalis* can colonize esophageal cancer tissues and infection in a mouse model of esophageal squamous cell carcinoma, the bacteria promote expansion of MDSCs in spleen (77).

Thus, *P. gingivalis* can modulate the microenvironment inflammatory promoting secretion of molecules which induce infiltration and expansion of MDSCs. Contrary to these reported observations, the systemic accumulation of M-MDSCs was decreased in a Familial Alzheimer’s disease mouse model infected with *P. gingivalis*, while that MDSCs or PNM-MDSCs did not show changes (79). Also, *P. gingivalis* reduced immunosuppressive capacity of M-MDSCs. It was suggested that these apparent discrepancies could be related with of age of analysed mice (79). Age of the mice used in the reports included in this section was around 6–12-week-old and the Familial Alzheimer’s disease mouse model was 20-week-old. Additionally, genetic background of Familial Alzheimer’s disease mice model is important, these mice are double transgenic overexpressing both mutant human amyloid precursor protein (APP) and mutant human presenilin (PS1); which results in rapid formation of amyloid plaque in brain, amyloid deposition begins at 2 months and the neuronal loss and brain dysfunction is exhibited at 4-5 months of age (79). Interestingly, *P. gingivalis* infection in the Familial Alzheimer’s disease mice model worsened neuroinflammation and the cognitive damage. While the treatment with exogenous M-MDSCs of the infected Familial Alzheimer’s disease mice model improved the cognitive function and reduced neuroinflammation (79). It is possible that different factors provoke changes in the composition of the inflammatory microenvironment resulting in variations of the MDSC biological behaviour (**Figure 3B**). Thus, particular inflammatory profiles generated by different diseases could modify functions of MDSCs, which can be exploited in the searching of possible treatments.

### 3.3 Streptococcus mutans

*S. mutans* is gram-positive microorganism considered a major etiological agent of dental caries and resides in the multispecies biofilm formed on hard surfaces of the tooth (80, 81). Potential cariogenic of *S. mutans* has been associated to the capacity to produce extracellular polymers of glucan allowing permanent colonization of hard surfaces and formation of the extracellular polymeric matrix, ability to prosper under low pH conditions and the ability to transport and metabolize a wide range of dietary carbohydrates to produce organic acids that demineralize tooth enamel. *S. mutans* also has been associated to other diseases as endocarditis (80, 81). Recently, it was suggested that this bacterium can promote more infiltration of MDSCs (CD11b^+^Gr-1^+^) into tumours in tongue, which was observed in an oral-tongue cancer mouse model infected with *S. mutans*. It was suggested that the bacteria increased the expression of IL-6 in tissues promoting the MDSC recruitment (**Figure 3C**) (82). More studies are required to determine the effect of *S. mutans* on biological functions of MDSCs. Interestingly, *S. mutans* does not act alone in the development of dental caries and can interact with other residents of oral cavity. For example, association with the yeast *Candida albicans* increases biofilm formation and cariogenicity (80). Therefore, to better understand the contribution of MDSCs in the context of oral cavity, studies examining the effects of biofilm on the biology of MDSC should be performed to complement the studies carried out using single-species microorganisms.

## 4 Myeloid-derived suppressor cells and *Candida* species

Species of the genera *Candida* are accountable for most infections caused by fungal pathogens in humans (83) and are commonly encountered in healthy individuals, being members of the normal oral microbiome (84). *Candida* genus included more than 150 species, of which only a few are medically important (85), such as *Candida albicans*, *Candida auris*, *Candida krusei*, *Candida tropicalis*, *Candida parapsilosis*, *Candida rugosa*, *Candida guilliermondii*, and *Candida glabrata* (85). *C. albicans* commonly colonizes the oral mucosa and is the most recovered specie of oral fungal isolates of healthy individuals (84). However, it can become an opportunistic invader causing fungal overgrowth and invasion of superficial tissues including tongue and other oral mucosal places. *C. albicans* is considered the major causative agent of oral candidiasis (86). Oral candidiasis can present multiple clinical presentations and can be classified in acute manifestations, chronic manifestations, and chronic mucocutaneous candidiasis syndromes (86).

**Figure 4:**
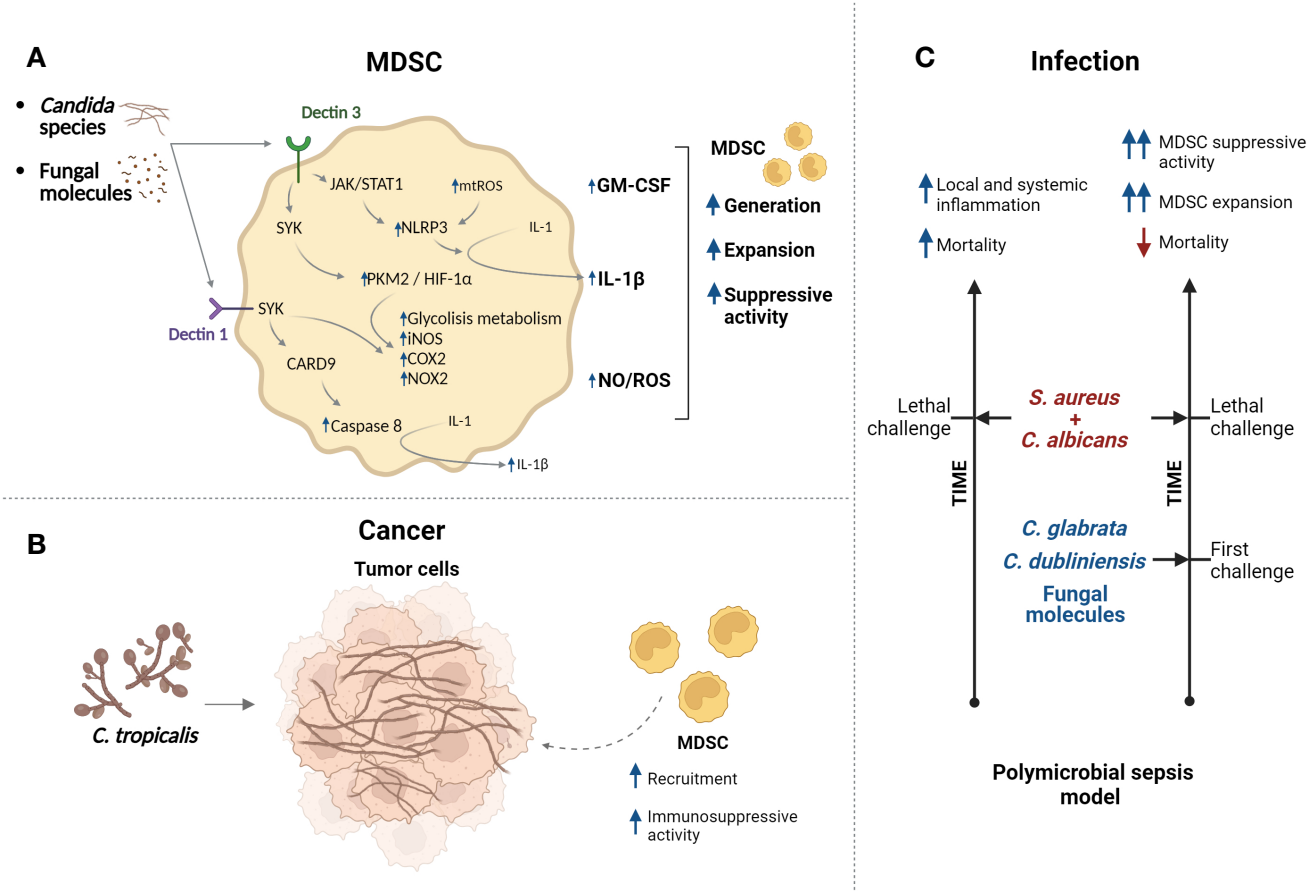
*Candida* species regulate functions of MDSCs. **(A)** *Candida* species/fungal molecules can be recognized by Dectin receptors which control different pathway (mechanisms are more described in *Section 4*) resulting in modulation of MDSC generation, expansion, and immunosuppressive activity. **(B)** In cancer context, infection with *C*. *tropicalis* promotes recruitment of MDSCs with augmented immunosuppressive activity. **(C)** Model of polymicrobial sepsis generated by infection with *C*. *albicans/S. aureus* (lethal challenge) present high mortality and inflammation. However, challenge with low-virulence *Candida* species or fungal molecules protects against lethal infection. It was suggested that expansion of MDSCs and their immunosuppressive activity participate in the protection. Created with BioRender.com.

The involvement of MDSCs in oral candidiasis or clinical presentations has not been addressed yet. However, studies analyzing the effect of species of the genera *Candida* on the MDSC biology have been reported. Transition capacity between yeast and hyphal morphotypes of *C. albicans* is fundamental in its pathogenesis allowing initial attachment, dissemination, and invasion of tissues (86). Analysis *in vitro* indicated that yeast and hyphal forms of *C. albicans* promoted generation and expansion of mouse and human MDSCs with high immunosuppressive activity (87, 88). Moreover, different *Candida* species promote *in vitro* generation and suppressive function of PMN-MDSCs differentially. *C. albicans* induces more generation of PMN-MDSCs, then *C. krusei* and *C. glabatra* and finally *C. parapsilosis* and *C. dubliniensis*. On the other hand, *C. krusei* and *C. glabatra* promote the generation of more immunosuppressive PMN-MDSCs that the PMN-MDSCs generated by *C. albicans* (88). This is interesting due to potential use of highly immunosuppressive MDSCs in adoptive transference as therapy. Moreover, other study reported that *C. tropicalis* and fungal molecules such as zymosan and mannans can promote MDSC and PMN-MDSC generation *in vitro* with immunosuppressive activity (89). Additionally, mice infected with *C. albicans* showed increased number of splenic PMN-MDSCs; also, PMN-MDSC expansion was observed in blood of immunosuppressed patients with invasive *Candida* bloodstream infection. Mechanistically, it was suggested that fungal components of *C. albicans* and other species are sensed by the receptor Dectin1 presents in MDSCs, this could activate tyrosine kinase Syk and promote function of domain family member 9 (CARD9) adaptor protein; this can result in generation of ROS, activation of caspase-8 and maturation of IL-1β (**Figure 4A**). Moreover, expression of receptor IL-1R by MDSCs permits auto-stimulation and incremented production of ROS and IL-1β, resulting in expansion of MDSCs (87). Besides, *Candida* species stimulate secretion of GM-CSF by MDSCs, which could promoter MDSC expansion (88).

On the other hand, specific mechanisms of regulation of MDSC suppressive functions by *C. tropicalis* have been proposed in the context of cancer. Oral treatments with *C. tropicalis* can promote recruitment of MDSCs in colon tissues of a mice model of colitis-associated colon cancer (89) supporting cancer development and aggravation of colitis (90). *C. tropicalis* molecules are sensed by Dectin3 in MDSCs, this promotes phosphorylation of Syk and its binding to Dectin3; then, Syk binds and phosphorylates tyrosine 105 residue of Pyruvate kinase M2 (PKM2) resulting in activation and nuclear translocation of PKM2 in MDSCs. Interaction of PKM2 and HIF-1α could promote the expression of glycolytic enzymes [Glut1, HK2, PKM2, LDHA and pyruvate dehydrogenase kinase 1 (PDK1)], Nos2 (iNOS), Ptgs2 (COX2), Cybb (NOX2) and more HIF-1α. Thus, *C. tropicalis* promotes generation of NO and ROS and induces MDSC metabolic reprogramming by the increase of Dectin3/PKM2/HIF-1α-dependent glycolytic metabolism, resulting in intensified immunosuppressive function of MDSCs (90). Importantly, *C. tropicalis* can induce more expression of Dectin3 in MDSCs (91), suggesting a positive feedback loop in activation of this pathway. Interestingly, the glycolytic reprogramming, induced by *C. tropicalis*, depended on activation of glycogen metabolism in MDSCs. Enhanced aerobic glycolysis levels, as well as the activation of JAK-STAT1 pathway via Dectin3 induced by *C. tropicalis* can promote the activation of the NLRP3 inflammasome in MDSCs resulting in secretion of IL-1β, which has been related with MDSC expansion. Additionally, it was suggested that *C. tropicalis* can promote generation of mitochondrial ROS in MDSCs and this contribute to more activation of NLRP3 inflammasome (91). Thus, *Candida* species can modulate generation, function, phenotype, and metabolism of MDSCs in cancer context (**Figure 4B**). Analyses of these effects in context of oral cavity in health could allow to understand possible contribution of MDSCs modulated by *Candida* species (as ubiquitous commensal microorganisms that normally colonize the oral mucosa) in maintenance of equilibrium between microbiota and immune system of host. Moreover, studies evaluating contribution of MDSCs in oral infections by *Candida* species (as opportunistic pathogens) could allow to assess potential therapeutic strategies.

Interestingly, the adoptive transference of PMN-MDSCs in a murine model of systemic *C. albicans* infection incremented percent survival. It was suggested that it was due to the reduction on activation of NK and T cells in kidney, decreased splenic Th17 cells and diminished levels of serum TNFα. Phagocytoses of *Candida* by MDSCs also was reported (87). Moreover, in a polymicrobial-intra-abdominal infection mouse model the coinfection with *C. albicans* and *Staphylococcus aureus* produced high mortality as consequence of a strong local and systemic inflammation. However, coinfection with *C. dubliniensis* and *S. aureus* or with other low-virulence *Candida* species (for example *C. glabrata* or *C. auris*) resulted in reduced mortality. Immunization with low-virulence *Candida* species or with *C. dubliniensis*/*S. aureus* conferred protection against lethal polymicrobial infection over a time period in post-changed mice with lethal *C. albicans*/*S. aureus* (**Figure 4C**). It was suggested that PMN-MDSCs could be responsible for this protection, which can be a form of trained innate immunity (nonspecific memory mediated by innate cells), and it was proposed to call “trained tolerogenic immunity” (92–95). Immunization with fungal molecules, such as β-glucans, also provide high level protection against lethal polymicrobial infection in mice models, which is mediated by PMN-MDSCs (96). Characterization of these cells showed that MDSC (CD11b^+^Gr1^+^) and PMN-MDSCs (CD11b^+^Ly6G^+^Ly6C^+/low^) cells are increased in bone marrow, spleen, blood, and peritoneal cavity in mice immunized with low-virulence *Candida* as *C. dubliniensis* and post-changed with lethal *C. albicans*/*S. aureus* compared with nonimmunized mice and post-changed with lethal *C. albicans*/*S. aureus*. Additionally, MDSC cells from immunized mice showed increased immunosuppressive capacity with increased expression of Arginase 1, INOS and NO production in comparison to MDSC cells from nonimmunized mice. It was suggested that PMN-MDSCs are required to provide protection against lethal polymicrobial sepsis involving a mechanism dependent on IL-10 production (97). Recently, it suggested that the induction of protection by MDSCs against lethal polymicrobial sepsis is better when the *Candida* species used in the immunization produce less damage to bone marrow tissue and cells (98).

The interaction fungi-host oral mucosa requires a fine-tuned balance between pro-inflammatory response and regulatory immune mechanisms. Alterations in this equilibrium could result in infections and disease; thus, studies analyzing presence, phenotypes and immunomodulate functions of MDSCs during the presence of the diverse species of *Candida* in the context of oral mucosa in health or disease could show potential therapeutic targets.

## 5 Discussion and conclusion

The second largest and diverse microbiota after the gut is found in oral cavity. This microbiota remains in a dynamic balance with the host and can colonize hard surface of the teeth and the soft tissues as oral mucosa. Microbiota and immune system maintain a bidirectional communication finely regulated and highly coordinated with the goal to preserve both local and systemic homeostasis resulting in host biological integrity (99). In this sense, different immunoregulatory cells are localized in oral cavity, MDSCs have been detected in oral structures such as submandibular glands (34), also M-MDSC and PMN-MDSC subsets with increased immunosuppressive capacity were found in alveolar (mandibular) bone in periodontal health (100); however, the contribution of MDSCs in the preservation of the homeostasis in oral cavity has not been clarified. As already described in previous sections, oral bacterial and fungal microorganisms can modulate functions of MDSCs; whether this modulation allows the maintenance of an appropriate equilibrium between the oral microbiota and host immune responses in health conditions and symbiosis has not been described. Most of the reported information focused on studying biological behavior of MDSCs when an imbalance between the microbiota and the host (dysbiosis) was present. Moreover, the information described comes from studies analyzing MDSC biological aspects in infection models using single-species microorganisms. Further studies analyzing the effects of symbiotic or dysbiotic microbiota on MDSCs could contribute to understand the complex process of immune regulation in oral cavity.

The periodontal disease in association with other inflammatory conditions or presence of periodontopathogens can induce systemic expansion of subpopulations of MDSCs and induce osteoclastogenic activity, which could be relevant in development of periodontal disease. However, the presence of MDSC subpopulations as osteoclast precursors should be analyzed in periodontal tissues. Furthermore, the involvement of MDSCs in human periodontitis and peri-implantitis remains to be determined experimentally. Interestingly, MDSCs could be implicated in the association between oral inflammatory disease/oral pathogens and systemic inflammatory diseases that have proved to induce MDSCs such as diabetes, cancer, rheumatoid arthritis, cardiovascular diseases or COVID-19 (11, 101–103), however this remains to be established. Further studies should analyze more deeply the involvement of MDSCs in other oral inflammatory diseases such as pulpitis, gingivitis, or mucositis. Additionally, it is possible that some oral microorganisms do not affect biological aspects of MDSCs. For example, *Streptococcus sanguinis*, a commensal specie, is found abundantly in the early oral biofilm and has been strongly associated with oral health (104). Oral administration of *S. sanguinis* in a mice model of intestinal tumorigenesis did not promote recruitment and infiltration of MDSCs in tumors in contrast with *F. nucleatum* treatments (59). Importantly, the effects were not determined in oral cavity. Thus, modulation of MDSC functions by additional oral microorganisms should be analyzed. MDSCs could be target of treatments in inflammatory oral diseases as it is suggested by the diverse experimental strategies used to ameliorate Sjögren’s syndrome symptoms or immunization with low-virulence *Candida* species. The changes in the inflammatory microenvironments generated in the diverse oral inflammatory diseases and by the oral pathogens promotes MDSC reprogramming, which could be valuable in the searching of experimental treatments.

## Author contributions

FG-A: Writing – original draft, Writing – review & editing. AL-M: Writing – original draft, Writing – review & editing. JG-R: Writing – original draft, Writing – review & editing. MI-E: Writing – original draft, Writing – review & editing. IS-H: Writing – original draft, Writing – review & editing. DF-R: Writing – original draft, Writing – review & editing. JV-J: Writing – original draft, Writing – review & editing. NS-H: Conceptualization, Funding acquisition, Writing – original draft, Writing – review & editing.
